# Accelerated Intermittent Theta Burst Stimulation (iTBS) for Crisis Stabilization in a Transgender Adolescent with Treatment-Resistant Depression: A Case Report

**DOI:** 10.7759/cureus.98115

**Published:** 2025-11-29

**Authors:** Connor Welz

**Affiliations:** 1 Research, The Mind Store, Lone Tree, USA

**Keywords:** adolescent depression, intermittent theta burst stimulation (itbs), neuromodulation therapy, recurrent suicidality, transgender health, treatment-resistant depression (trd)

## Abstract

Transgender adolescents experience disproportionately high rates of depression and suicidality, yet effective interventions for those unresponsive to conventional treatments remain scarce. This case report describes the case of a 15-year-old transgender male with severe treatment-resistant depression and active suicidal ideation who underwent an accelerated intermittent theta burst stimulation (iTBS) protocol: 20 sessions over four consecutive days targeting the left dorsolateral prefrontal cortex. Baseline scores indicated severe depression (Patient Health Questionnaire-9 (PHQ-9) = 22) and high suicide risk (Suicidal Ideation Attributes Scale (SIDAS) = 35). At the one-month follow-up, the patient's symptoms improved to minimal depression (PHQ-9 = 4) and below the high-risk suicide cutoff (SIDAS = 10). He reported improved mood, reduced suicidal thoughts, and no adverse effects. To the author's knowledge, this is the first published case of accelerated iTBS in a transgender adolescent demonstrating rapid and substantial symptom reduction with good tolerability. These findings suggest accelerated iTBS may be a safe and time-sensitive option for crisis stabilization in transgender youth when traditional approaches fail, warranting further research in larger cohorts.

## Introduction

Major depressive disorder (MDD) and suicidality remain leading causes of adolescent disability and death worldwide [1]. Transgender adolescents experience significantly elevated rates of suicidal ideation and attempts compared to cisgender peers [2-4]. Furthermore, those who do not respond to traditional treatments are at a higher risk of suicide [5]. Intermittent theta burst stimulation (iTBS) is a non-invasive brain stimulation technique targeting the dorsolateral prefrontal cortex (DLPFC). While iTBS has demonstrated efficacy and good tolerability in treatment-resistant adult depression [6], its efficacy, safety, and tolerability, particularly using accelerated protocols, remain unstudied in adolescents experiencing treatment-resistant depression compounded by gender minority stress, with no published studies focusing on transgender youth [7].

Here, we present the case of a 15-year-old transgender male with severe treatment-resistant depression and active suicidal ideation who received an accelerated iTBS protocol for crisis stabilization. Accelerated iTBS protocols deliver multiple sessions per day over a condensed timeframe and differ from standard transcranial magnetic stimulation (TMS) by substantially increasing the daily treatment dose while maintaining brief session durations. These accelerated protocols aim to induce rapid clinical improvement.

Objectives

This case report presents three primary objectives. First, it aims to document the novel application of the iTBS protocol in a high-acuity demographic: a transgender adolescent with treatment-resistant depression and active suicidal ideation. Second, it rigorously examines this high-frequency intervention's clinical efficacy and safety profile (five sessions/day × four days) within this single-subject context, with particular attention to its potential for crisis stabilization. Third, a quantitative evaluation of symptom trajectories is conducted using prospectively administered, standardized instruments, such as the Patient Health Questionnaire-9 (PHQ-9) [8] for depressive severity and the Suicidal Ideation Attributes Scale (SIDAS) [9] for acute suicide risk. Finally, this work explores the tolerability and feasibility of accelerated iTBS regimens in adolescent populations, a critical gap in neuromodulation literature [10-11] with implications for scaling rapid interventions in developmental cohorts facing intersectional vulnerabilities.

Ethical considerations

This case report adheres to the guidelines for Case Reports (CARE) and the ethical principles outlined in the Declaration of Helsinki, and as it constitutes a retrospective analysis based on de-identified clinical data for educational and quality improvement purposes, it is deemed exempt from requiring full Institutional Review Board (IRB) approval. Before treatment initiation and the anonymized publication of clinical outcomes, written informed consent was obtained from the adolescent patient. Additionally, written informed consent was secured from the patient's legal guardians (parents).

## Case presentation

The patient is a 15-year-old transgender male whose psychiatric diagnoses were established through a comprehensive clinical assessment per the Diagnostic and Statistical Manual of Mental Disorders, fifth edition (DSM-5) [12]. The diagnoses include major depressive disorder, single episode, severe without psychotic features (ICD-10: F32.2); generalized anxiety disorder (F41.1); generalized social anxiety disorder (social phobia; F40.11); and post-traumatic stress disorder, unspecified (F43.10). His prior pharmacologic treatment included one SNRI trial and one SARI trial, each yielding only partial symptomatic improvement without achieving remission.

Presentation and patient history

The patient reported a history of frequent geographic relocations during childhood secondary to his father's service in the United States Air Force. He described significant familial difficulty accepting his gender identity, contributing substantively to his experience of emotional rejection and lack of support. Prior therapeutic interventions included multiple trials of antidepressant medications, which yielded only partial symptomatic relief without achieving sustained remission. Concurrently, the patient engaged in psychotherapy targeting his mood, anxiety, and trauma symptoms. The chronicity and complexity of these co-occurring psychiatric conditions were assessed as significant contributors to his high baseline symptom severity upon presentation.

Treatment protocol

The patient underwent an accelerated course of TMS utilizing the iTBS protocol using a Brainsway Deep Transcranial Magnetic Stimulation (dTMS) system with an H1 coil (BrainsWay Ltd., Jerusalem, ISR) [13]. Treatment consisted of five iTBS sessions per day, administered over four consecutive days, resulting in 20 treatment sessions. This intensive schedule was based on emerging evidence for accelerated protocols in treatment-resistant conditions [14].

Stimulation targeted the DLPFC using the BrainsWay standard iTBS parameters for major depressive disorder [15]. This included bursts of three 50 Hz pulses delivered at 5 Hz, with 10 bursts (30 pulses) per two-second train, an eight-second inter-train interval, 20 trains per session, and 600 pulses total (~3 minutes/session). The motor threshold (MT) was determined via visible right thumb twitches in ≥5/10 trials with the coil tangentially positioned over the left primary motor cortex. The DLPFC was localized 5.5 cm anterior to the motor site along a parasagittal line [16], with intensity set at 80% MT. Adverse effects were monitored through open-ended questioning after each session and formal assessment at the one-month follow-up. No alterations to pharmacotherapy or psychotherapy were permitted during treatment or the four-week follow-up period to isolate iTBS effects.

Outcome measures

Depressive symptom severity was quantified using the PHQ-9, a nine-item self-report instrument validated for assessing depression severity in adolescents [8]. Acute suicide risk was evaluated via the SIDAS, a validated five-item scale measuring frequency, controllability, closeness to attempt, and distress associated with suicidal thoughts [9]. Both standardized instruments were administered at baseline (pre-treatment) and at the one-month follow-up to track longitudinal changes.

Results

The patient reported improved mood, reduced suicidal thoughts, better emotional regulation, increased social engagement, and no adverse effects. Adverse effect monitoring was conducted via patient self-report; no headaches, scalp discomfort, or other stimulation-related side effects were reported. Quantitative assessment (Table 1) revealed substantial clinical improvement across primary endpoints. Depressive symptom severity, measured by the PHQ-9, demonstrated a marked reduction from a baseline score of 22 (indicating severe depression) to 4 (minimal range) one month post-treatment, reflecting a clinically significant response. Similarly, acute suicide risk quantified via the SIDAS decreased from 35 (high risk) to 10 (low risk) at the same follow-up interval. A longitudinal treatment timeline visually contextualized this rapid trajectory, charting daily progression across the four-day accelerated iTBS protocol (20 sessions total) with annotated outcome milestones confirming symptom reduction initiated during active treatment and sustained through follow-up.

**Table 1:** Pre- and post-treatment scores on the PHQ-9 and SIDAS PHQ-9: Patient Health Questionnaire-9, SIDAS: Suicidal Ideation Attributes Scale

Measure	Baseline score	Post-treatment score	Clinical interpretation
PHQ-9	22/27 (severe)	4/27 (minimal)	Severe → Minimal
SIDAS	35 (high risk)	10 (below high risk)	High risk → Below high-risk cutoff

## Discussion

This case illustrates a rapid and dramatic improvement in depressive symptoms and suicidal ideation following an accelerated iTBS protocol. The intervention was well-tolerated, with no adverse events, suggesting a potential role for iTBS in treatment-resistant adolescent populations [17]. In clinical practice, accelerated iTBS may be considered a feasible option for rapid stabilization in adolescents when hospitalization is not possible or traditional treatments have been exhausted, though controlled trials are needed before broad implementation. 

Potential neurobiological mechanisms in adolescents

Gamma-aminobutyric acid (GABA) and glutamate are neurotransmitters regulating inhibitory and excitatory signaling in the brain [18]. Developmental variations in these systems within emotion-regulation regions during adolescence may contribute to heterogeneity in depressive symptom presentation and differential treatment responsiveness [19], potentially downregulating limbic hyperactivity in adolescents with immature frontal inhibition. The iTBS may enhance synaptic plasticity in the dorsolateral prefrontal cortex via modulation of glutamatergic and GABAergic systems. This mechanism is particularly relevant for adolescent depression, where developmental differences in frontolimbic GABAergic and glutamatergic tone may explain variations in symptom profiles and treatment response. A deeper understanding of these neurochemical dynamics, measurable via TMS, could advance precision medicine and targeted interventions.

Intersection with gender minority stress

Transgender adolescents often experience chronic minority stress, involving sustained exposure to stigma, discrimination, and identity invalidation [20]. These stressors can contribute to dysregulation of limbic-prefrontal circuitry [21]. Neuromodulation may offer a pathway to reduce hyperactivity in threat-related networks and enhance top-down emotional regulation.

Comparison with existing literature

While pediatric TMS studies suggest efficacy for treatment-resistant depression, to the author's knowledge, there are no published case studies specifically involving transgender adolescents receiving iTBS. This case adds to a sparse but growing area of inquiry.

Clinical applicability

In real-world adolescent care, accelerated iTBS may be considered as a rapid stabilization option for treatment-resistant depression, particularly in high-risk youth when immediate hospitalization is not feasible. Clinicians should be aware of potential risks, including discomfort, headaches, or rare seizure events, and ensure careful monitoring during and after treatment. Additionally, reproducibility may be influenced by factors such as precise coil placement, stimulation parameters, and patient adherence, underscoring the need for standardized protocols in clinical practice.

Limitation beyond sample size

The novelty and intensity of the treatment protocol may have amplified the potential influence of placebo effects. Additionally, the short follow-up period (one month) restricts conclusions about the durability of clinical improvements over time. The one-month follow-up limits durability assessment; future studies should include three- to six-month evaluations. Finally, adverse effects were assessed solely through participant self-report, without using a standardized adverse event checklist, which may limit the completeness and accuracy of safety monitoring. Reports of adverse events were based on self-report; standardized tools (e.g., TMS safety checklist [22]) would enhance objectivity.

## Conclusions

To this author’s knowledge, this represents the first documented application of accelerated iTBS in a transgender adolescent, suggesting the possible feasibility of this approach for rapid crisis stabilization in high-acuity populations. The intervention yielded clinically significant reductions in both depressive symptoms (PHQ-9: 22→4) and acute suicide risk (SIDAS: 35→10) within one month, indicating a potentially meaningful short-term clinical response. Accelerated iTBS was well-tolerated with no serious adverse events, supporting its consideration as a time-sensitive intervention for suicidal transgender youth when conventional therapies (pharmacotherapy, psychotherapy) prove inadequate. However, the generalizability of these findings is inherently limited by the single-case design, and no causal conclusions can be drawn. These results should be viewed as preliminary and hypothesis-generating, underscoring the need for larger controlled trials with extended follow-up to validate efficacy, optimize parameters, and establish safety profiles in demographically diverse adolescent cohorts.
